# O.6.1-1 Knowledge and awareness of physical activity among Irish adolescents: findings from a whole-of-school programme

**DOI:** 10.1093/eurpub/ckad133.260

**Published:** 2023-09-11

**Authors:** Caera L Grady, Kwok Ng, Elaine Murtagh, Catherine B Woods

**Affiliations:** Physical Activity for Health Research Centre, Health Research Institute, University Of Limerick, Ireland; Department of Physical Education and Sports Science, University of Limerick; Physical Activity for Health Research Centre, Health Research Institute, University Of Limerick, Ireland; Department of Physical Education and Sports Science, University of Limerick; School of Educational Sciences and Psychology, University of Eastern Finland; Faculty of Education, University of Turku, Finland; caera.grady@ul.ie; Physical Activity for Health Research Centre, Health Research Institute, University Of Limerick, Ireland; Department of Physical Education and Sports Science, University of Limerick; Physical Activity for Health Research Centre, Health Research Institute, University Of Limerick, Ireland; Department of Physical Education and Sports Science, University of Limerick

## Abstract

**Purpose:**

Adolescents are among the least physically active population sub-groups. Adolescence is a key transition period in which physical activity (PA) habits develop and track into adulthood. Knowledge and awareness are key factors of behaviour change. Yet, few adolescents can correctly identify the recommended PA guidelines to achieve health benefits. In Ireland, the second-level Active School Flag (SLASF) programme is an example of a whole-of-school programme (https://activeschoolflag.ie/). This study aimed to examine the change in adolescents’ levels of knowledge and awareness of PA at the beginning at end of year one of a whole-of-school programme.

**Methods:**

A repeated measures study design with a questionnaire administered in five SLASF schools during Nov 2021 and May 2022 (N = 301, 60.5% male, Mean age=15.5y± 1.82). Items from the determinants of PA questionnaire (DPAQ), health literacy 10-item questionnaire, programme specific variables relating to knowledge and awareness of SLASF, identifying the correct recommended PA guidelines and self-reported moderate-vigorous PA (MVPA) were included. A paired t-test or Wilcoxon-signed ranked test were run to determine mean differences over time for DPAQ- knowledge and beliefs about consequences, health literacy, the ability to report the recommended PA guidelines over time and self-reported PA levels. A chi-squared test was used for the programme related categorical variables.

**Results:**

Adolescents MVPA increased from November (M = 3.82±1.92) to May (M = 3.96±1.9) *(t(389) = -1.446, p<.001)*. There was an improvement in adolescents knowing the reasons for meeting the guidelines *(Z= -4.113, p<.001)* from Nov (M = 5.05±1.59) to May (M = 4.48±1.89). Furthermore, there was an increased previous awareness of the programme from Nov (M=.63±.67) to May (M=.77±.63) *(X*^*2*^*(4, N = 296) = 62.96, p<.001)*. Participants awareness of the programme leaders in the school increased over time: Staff (M=.35±.48) to (M=.42±.5) *(X*^*2*^*(1, N = 197) = 37.7, p<.001)*, Students (M=.32±.47) to (M=.36±.48) *(X*^2^*(1, N = 204) = 21.35, p<.001).*

**Conclusions:**

Across the year, participants in SLASF schools became more physically active. Some aspects of knowledge and awareness of both PA and the SLASF programme showed promising improvements however, these participants are in the early stages of the SLASF programme thus, it would be beneficial to observe these changes over a longer period of time.

